# Sunitinib: An Unusual Cause of Pneumothorax in a Patient With Metastatic Chromophobe Renal Cell Carcinoma

**DOI:** 10.7759/cureus.9586

**Published:** 2020-08-06

**Authors:** Nedal Bukhari, Suha Al-Badr, Manal AlNaimi, Faisal Azam

**Affiliations:** 1 Medical Oncology, King Fahad Specialist Hopsital, Dammam, SAU; 2 Internal Medicine, Imam Abdulrahman Bin Faisal University, Dammam, SAU; 3 Medical Imaging, King Fahad Specialist Hospital, Dammam, SAU; 4 Surgery/Thoracic Surgery, King Fahad Specialist Hospital, Dammam, SAU; 5 Medical Oncology, King Fahad Specialist Hospital, Dammam, SAU

**Keywords:** sunitinib, vascular endothelial growth factor receptor, pneumothorax, renal cell carcinoma

## Abstract

Spontaneous pneumothorax secondary to sunitinib, a vascular endothelial growth factor receptor (VEGFR) inhibitor, is an extremely rare side effect of this class of medications. In this report, we present the case of a patient with metastatic renal cell carcinoma (RCC) who developed bilateral pneumothoraces after starting on sunitinib. This case report recognizes pneumothorax as a life-threatening side effect of sunitinib.

## Introduction

Sunitinib was the first tyrosine kinase inhibitor (TKI) approved for the treatment of clear cell renal cell carcinoma (RCC) based on the results of a phase III trial [1,2]. It is also approved for treating metastatic non-clear cell RCC, including the chromophobe subtype [3]. Common side effects of sunitinib are fatigue, nausea, diarrhea, hypertension, and hand and foot syndrome. Patients on sunitinib may also experience other gastrointestinal, cardiovascular, dermatological, hematological, and respiratory side effects [1,2,4]. Pneumothorax secondary to sunitinib has been reported previously in two patients with metastatic clear cell type RCC [5,6]. To our knowledge, this is the first study to report a case of metastatic chromophobe RCC experiencing pneumothorax secondary to sunitinib [5,6].

## Case presentation

**Figure 1:**
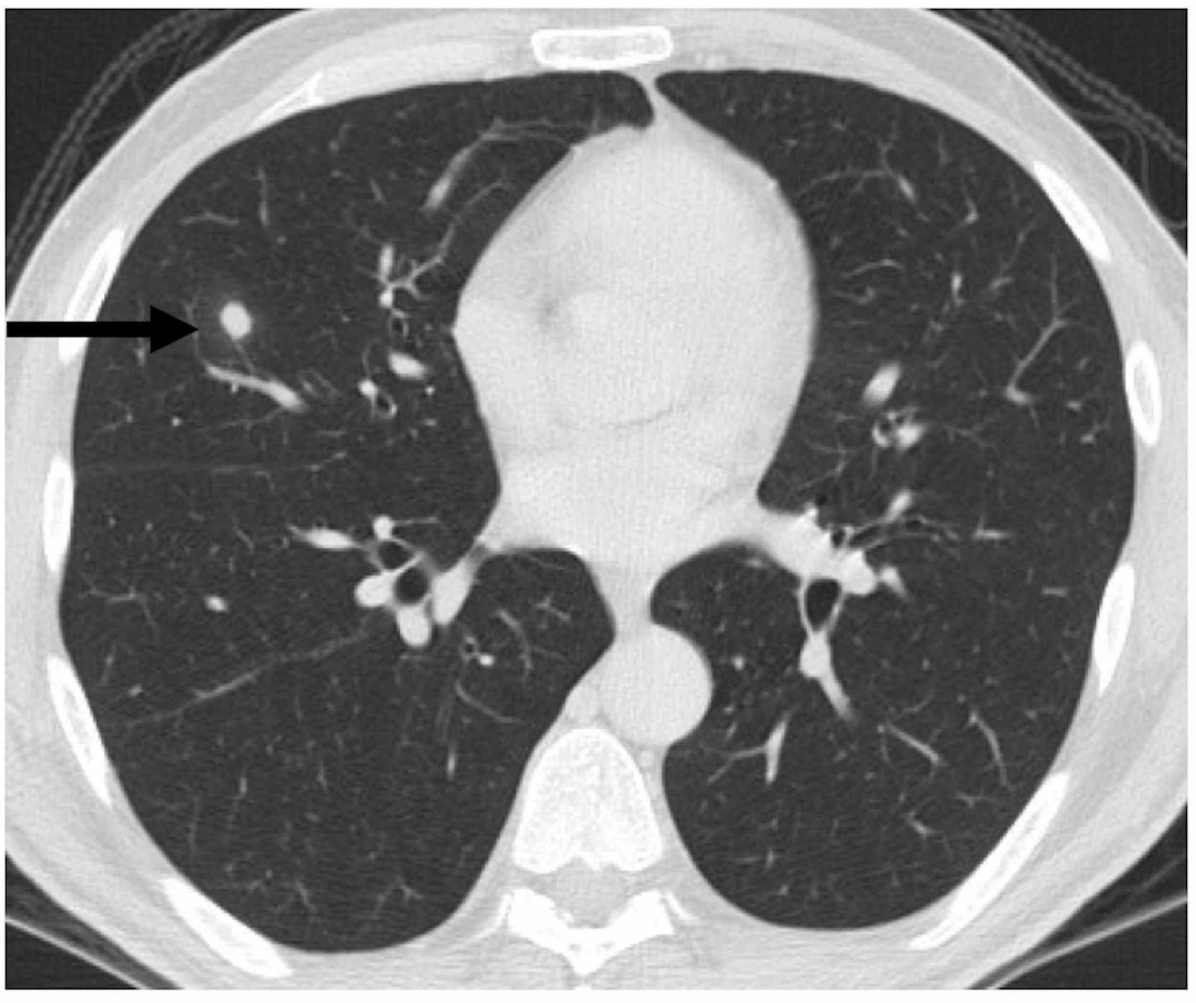
Enhanced axial thin-section chest CT (lung window) The image demonstrates a single right middle lobe round well-defined solid nodule (arrow), considered as newly developed pulmonary metastasis, i.e., disease progression CT: computed tomography

A 48-year-old male with a history of a left kidney mass consistent with renal cancer underwent a left radical nephrectomy with a pathology remarkable for grade IV chromophobe RCC with extensive sarcomatoid and rhabdoid features. Two out of eight lymph nodes were involved with the disease. His cancer was pathologically staged as T3N1 disease. Staging CT scans of the chest, abdomen, and pelvis (CAP) ruled out metastasis. Three months after his surgery, his CT CAP showed extensive retroperitoneal (RTPN) lymphadenopathy; he also had a right middle lobe (RML) lung nodule measuring almost 1 cm, all of which were radiologically consistent with metastatic disease (Figure 1).

**Figure 2:**
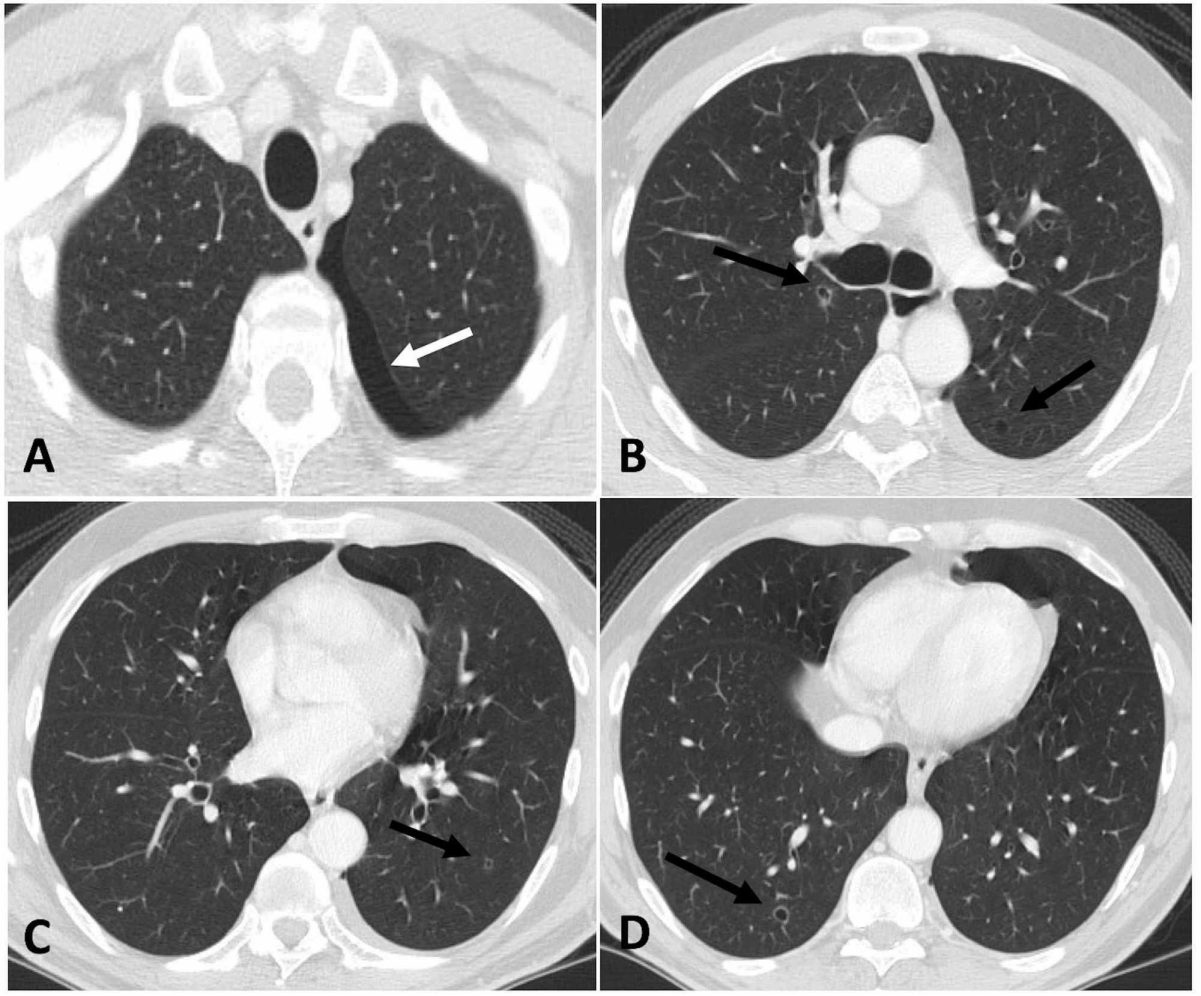
Enhanced CT at three-month follow-up (lung window) The images show multiple round thin wall cysts (black arrows in B, C, and D) and left pneumothorax (white arrow in A) CT: computed tomography

The patient was started on a modified three-weekly regimen of sunitinib, where he received a daily dose of 25 mg (two weeks on and one week off), with a plan to escalate the dose gradually by 12.5 mg in subsequent cycles until it reached 50 mg daily. A repeat CT CAP was done after four cycles of three-weekly sunitinib course and showed an interval regression in his metastatic RTPN lymphadenopathy and RML lung nodule. However, it showed a small left pneumothorax with the greatest diameter measured at 1 cm. Multiple bilateral cavitary lesions were noted as well (Figure 2).

**Figure 3:**
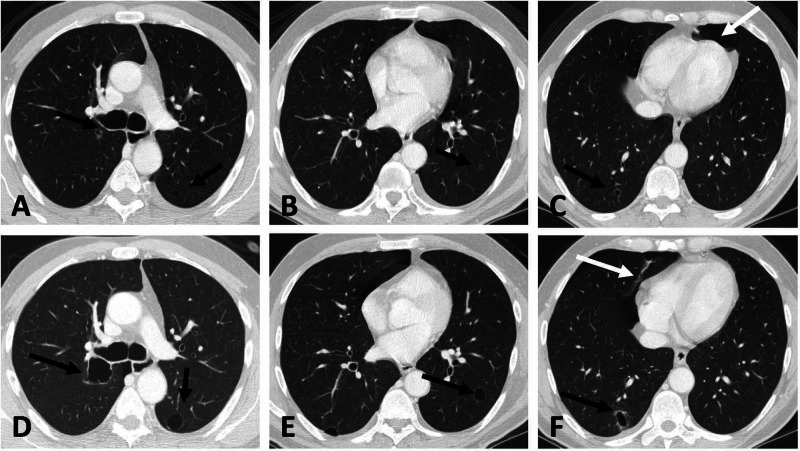
Enhanced CT at six-month follow-up (lung window) The thin wall cysts demonstrate enlargement in size exceeding 50% in volume and increase in wall thickness exceeding 3 mm in thickness, becoming cavitary in nature (images A, B, and C at three months; images D, E, and F at six months). Left pneumothorax completely resolved and a new pneumothorax developed on the right side (white arrow) CT: computed tomography

He was asymptomatic on review, and pneumothorax was treated conservatively with follow-up chest X-rays indicating its stability. Therefore, sunitinib was continued as per treatment plan. While approaching the end of the cycle eight, the patient developed sudden-onset shortness of breath that prompted an urgent chest X-ray followed by a repeat CT CAP, which showed persistence of the small left-sided pneumothorax and the appearance of a new right pneumothorax. Further regression in the RTPN lymphadenopathy and lung nodules was noted (cystic and cavitary lesions increased in number). Thoracic surgery service was consulted, and a right chest tube was inserted, which led to an immediate improvement in his symptoms. The left pneumothorax was treated conservatively (Figure 3).

The patient had no history of chronic lung diseases or trauma; he had never smoked and was not taking any other medications. We elected to stop his sunitinib and switch him to second-line nivolumab immunotherapy a few weeks after this event. Regular follow-up in the clinic and chest X-rays did not show any further pneumothoraces.

## Discussion

RCC is the 14th most common cancer worldwide with clear cell RCC being the most common variant of it [7]. Chromophobe subtype of RCC is a rare condition, representing only 5% of RCC cases and treated similarly [3,7]. RCC is a hyper-vascularized tumor with angiogenesis being essential for its growth, which is mainly mediated by vascular endothelial growth factor (VEGF). TKIs targeting VEGF have been the mainstay of treatment for over a decade [2,8].

Sunitinib is an orally administered anti-VEGF agent; it inhibits multiple tyrosine kinases, including vascular endothelial growth factor receptor (VEGFR), platelet-derived growth factor receptor (PDGFR), glial cell line-derived neurotrophic factor receptor (rearranged during transfection; RET), stem cell factor receptor (KIT), FMS-like tyrosine kinase-3 (FLT3), and the receptor for macrophage colony-stimulating factor (CSF-1R) [8,9].

The phenomenon of pneumothorax as an adverse drug event is well described in lung cancer patients receiving chemotherapy and antiangiogenic treatments, and it is usually preceded by cavitation [10,11]. It is also reported in patients with different solid tumors with lung metastasis after receiving antiangiogenics like bevacizumab, apatinib, pazopanib, sorafenib, regorafenib, and axitinib [10-14]. The mechanism of pneumothorax in patients on anti-VEGF medications is not well understood. However, many theories were hypothesized previously. The most likely mechanism in our case was tumor necrosis of subpleural nodules secondary to sunitinib, leading to fistula formation between the bronchus and the pleural cavity resulting in pneumothorax formation [5,11,15].

## Conclusions

Based on our experience of this case and previous cases reporting sunitinib-induced pneumothorax, we believe clinicians should be observant of new-onset respiratory symptoms in patients on this medication. And if pneumothorax is present, we recommend discontinuing this antiangiogenic agent and considering a different type of treatment.
